# Prevalence of IBS and its association with academic stress and dormitory lifestyle among medical students of Bangladesh: A cross-sectional study

**DOI:** 10.1016/j.heliyon.2024.e36259

**Published:** 2024-08-13

**Authors:** Tirthom Das, Fahmida Hoque Rimti, Hossain Ahmed Fahid, Nabil Ahmed Uthso, Mohammad Delwer Hossain Hawlader

**Affiliations:** a Department of Public Health, North South University, Dhaka, 1229, Bangladesh; b Chittagong Medical College, Chattogram, 4203, Bangladesh; c Institute of Statistical Research & Training, University of Dhaka, Bangladesh

**Keywords:** IBS, Stress, Medical students, Dormitory, Bangladesh

## Abstract

**Introduction:**

Irritable Bowel Syndrome (IBS) is a chronic gastrointestinal disorder affecting a substantial portion of the global population. While the prevalence of IBS is well-documented worldwide, limited research has explored its occurrence and associated factors among medical students in Bangladesh, a population exposed to high academic stress. This cross-sectional study aimed to assess the prevalence of IBS among medical students and investigate its potential association with stress levels and the dormitory lifestyle.

**Methods:**

Data were collected from 402 medical students using a self-administered questionnaire covering sociodemographic information, academic stress, lifestyle factors, and the Rome III Criteria for diagnosing IBS. Statistical analysis included bivariate and logistic regression analyses to identify significant associations and predictors of IBS prevalence.

**Results:**

This study among 402 university students found an overall irritable bowel syndrome (IBS) prevalence of 22.88 %, with 35.87 % diarrhea-predominant, 26.08 % constipation-predominant, and 38.04 % mixed subtype. Hostel residents had 2.11 times higher adjusted odds of IBS (95 % CI: 1.05–4.25, p < 0.001) than non-residents. IBS prevalence increased from 20.25 % for <1 year to 24.24 % for 1–3 years and 29.13 % for >3 years of hostel stay. Age 23–28 years (OR = 1.86, p = 0.030), lack of senior support (OR = 2.36, p = 0.05), second study phase (OR = 2.43, p = 0.002), inadequate exercise (OR = 2.11, p = 0.036), and frequent fatty food intake (OR = 2.98, p = 0.03) increased IBS risk. Higher academic stress (OR = 2.03, p = 0.002) predicted IBS, with 54.44 % vs. 43.78 % (p = 0.035) high stress among hostel residents who exercised less (48.23 % vs. 51.77 %) and consumed more fatty foods (53.33 % vs. 46.67 %). Mediation analysis revealed dormitory living impacts stress, physical activity, and diet - established IBS risk factors.

**Conclusion:**

The high prevalence of IBS among medical students in Bangladesh highlights the need for interventions to address changeable factors like academic stress, dormitory living conditions, lack of physical activity, and unhealthy eating habits to improve their health and wellness.

## 1 Introduction

Irritable bowel syndrome (IBS) is a chronic functional gastrointestinal illness marked by repeated bouts of stomach pain, discomfort, and bowel irregularities that are not explained by anatomical or biochemical abnormalities [1]. IBS is the most prevalent chronic disease affecting the digestive system, affecting 10–20 % of the global population [2]. Gender, dietary regimen, lifestyle, stress, post-traumatic stress disorder, and psychiatric disorders have all been mentioned as biological, psychological, and social factors in the pathogenesis of IBS [[3], [4], [5]].

According to a study of the European population, the overall prevalence of IBS was found to be 11.5 percent. These figures were extremely close to those reported in the United States [6], although in Asian countries, a wide range of 2.3–34 percent has been observed, and morbidity is on the rise [[7], [8], [9]]. Irritable bowel syndrome (IBS) commonly affects adults in their twenties and thirties and is associated with psychological anguish, sexual dysfunction, and job and sleep disruption [10]. To provide a complete scenario of such a chronic disease, health-related quality of life is an essential assessment tool [11]. When compared to healthy controls, patients with IBS have a lower quality of life on every scale using SF-36 [[12], [13], [14]].

Medical students with IBS have significantly higher stress scores and stressful life events than the non-IBS group [15]. IBS is significantly associated with a decrease in the academic performance of medical students [16]. Lack of exercise is also a significant predictor of IBS [17]. A higher prevalence of IBS is observed among medical students who were under constant stress, consumed alcohol, or used NSAIDs frequently and among those who had a family history of IBS [18]. A high prevalence of IBS prevailed among medical students and interns with Female gender, morbid anxiety, living in school dormitories, emotional stress, and higher educational level (grade) [19]. As a result, medical students are more likely to develop IBS than students in other disciplines. Studies also reveal a higher rate of IBS among students residing in dormitories than at home [20,21].

Despite the subject's relevance, more research needs to be undertaken in our community, particularly for medical students exposed to stressful environments during their medical school years. Medical education is known to be highly demanding, and stress-related disorders, such as IBS, can significantly impact the academic and overall well-being of students. Additionally, the unique dormitory lifestyle prevalent among many Bangladeshi medical students may have distinct influences on their health, making exploring its potential connection with IBS essential. Therefore, the objectives of this cross-sectional study in Bangladesh are to assess the prevalence of Irritable Bowel Syndrome (IBS) among medical students and investigate its potential association with stress levels and the dormitory lifestyle.

## 2 Methodology

### 2.1 Study design and participants

Utilizing a cross-sectional research design, data for this study were gathered from 402 medical students. The target population for this research consisted of medical students residing in Bangladesh. A convenience type of non-probability sampling technique was utilized to select participants, ensuring accessibility and practicality in data collection. The data collection procedure commenced in September 2022 and concluded in February 2023. A self-administered questionnaire was employed for data collection. Data was collected through a 4-person team of volunteers across Bangladesh. Before data collection began, the questionnaire underwent a pre-testing phase to assess its validity and reliability (Crohnbach's alpha: 0.73).

To streamline data gathering, we opted for a digital approach. We provided our targeted participants with a Google form, allowing each unique Internet Protocol (IP) address to submit just one response. The form was structured sequentially, with participants required to give their consent by selecting “Yes” before proceeding to subsequent sections. Throughout this process, we took rigorous measures to uphold our participants' anonymity and confidentiality. Their identities were meticulously shielded, ensuring their privacy and maintaining robust data security. We implemented data encryption and coding to preserve data integrity and facilitate secure storage and analysis. This study's protocol received approval from the institutional review board (IRB)/ethical review committee (ERC) of North South University under reference #2022IOR-NSU/lRB/1203.

### 2.2 Research instrument

A questionnaire for Sociodemographics and other variables was used to study the prevalence of IBS and its association with stress among medical students in Bangladesh. A validated, self-administered, semi-structured questionnaire was used for data collection. It contained questions regarding personal information, social characteristics, lifestyle and habits, family history of IBS, nutritional history, medication history, academic stress, and Rome III Criteria [22,23].

### 2.3 ROME III criteria

The diagnosis of IBS depended on the English version of “Rome III Criteria.” IBS, according to “Rome III Criteria,” is described as the clinical condition with recurrent abdominal discomfort or pain for at least three days/month, and a period of this discomfort has been reported in the past three months. The IBS is associated with some features (two or more of the following described features):

(a) Improvement in the condition with defecation,
(b) Onset of this condition related to changed frequency of stool,
(c) a change in the appearance or form of stool with the onset of IBS [24].

We used Rome III criteria to diagnose IBS even though Rome IV criteria were available during our data collection phase. This criterion allowed comparisons with epidemiological studies on IBS prevalence that used Rome III criteria. Using the same diagnostic criteria lets us more accurately compare our findings to the IBS prevalence study. This is crucial for Bangladesh and other South Asian communities because Rome IV data is scarce. The Rome III criteria have been widely used and confirmed in population-based studies, making them our study's benchmark.

### 2.4 Dormitory lifestyle

“Dormitory lifestyle” was operationalized as the living conditions and experiences associated with residing in on-campus student housing facilities (hostels) during medical education. To comprehensively assess this factor, the questionnaire included a set of questions covering various aspects of dormitory living. First, the living situation and duration of dormitory residence were determined. Questions on the frequency of outside food consumption and perceptions of food quality followed this. Finally, perceived support systems were assessed by examining support from seniors and peers.

### 2.5 Perception of Academic Stress Scale

Perceptions of Academic Stress Scale (PAS) is an 18-item, five-point Likert-type scale to measure perceptions, academic stress, and its sources in students [25]. This scale was standardized for students pursuing undergraduate and post-graduation studies. The responses ranged from strongly disagree [1] to strongly agree [5], measuring four dimensions with internal consistency; these dimensions are as follows: pressures to perform (0.6), perceptions of workload and examinations (0.6), self-perceptions (0.5) and time constraints (0.6). The overall internal consistency reliability was more than 0.7, with high scores indicating higher stress experienced by students. A score ranging from 25 to 40 would be considered low stress, while a score ranging from 41 to 60 was regarded as moderate stress, and scores upwards of 60 are considered severe stress [23].

### 2.6 Statistical analysis

Analysis was done with IBM SPSS Statistics 23.0 and R 4.2.2. Sociodemographic features, IBS subtypes, and other relevant variables were descriptively analyzed. This data comprised frequencies and percentages. Averages and standard deviations were used for continuous variables like age and stress scores. IBS prevalence was determined by the rate of subjects meeting Rome III diagnostic criteria. Statistical analyses examined the association between IBS prevalence and dormitory living, academic stress, lifestyle behaviors, and sociodemographic parameters. This was done using bivariate analyses, such as chi-square tests for categorical variables and independent t-tests or ANOVA for continuous variables. Multivariate logistic regression analysis uncovered IBS predictors while controlling for confounding variables. The study estimated odds ratios (ORs) and 95 % confidence intervals (CIs) for association magnitude. To explore the indirect effects of dormitory living on IBS, stress, physical activity, and diet were analyzed using mediation analysis. All analyses were statistically significant if p < 0.05.

## 3 Results

### 3.1 Sociodemographic characteristics

The study included 402 participants, of which 57.96 % were aged 17–22 years and 56.97 % were female. The majority (59.45 %) belonged to the middle socioeconomic status, and 74.63 % resided in urban areas. Most participants (94.03 %) were enrolled in the MBBS degree program (Table 1).

**Table 1:** Background characteristics of respondents.

Variables	Frequency (n)	Percentage (%)
**Age** 17–22 23–28	233 169	57.96 42.04
**Sex** Male Female	173 229	43.03 56.97
**Family socioeconomic status** Lower or lower-middle Middle Upper or upper-middle	55 239 108	13.68 59.45 26.87
**Type of residence** Rural or semi-urban Urban	102 300	25.37 74.63
**Degree enrolled** BDS MBBS	24 378	5.97 94.03
**Study phase** 1st phase 2nd phase 3rd phase 4th phase	140 73 86 103	34.83 18.16 21.39 25.62
**Ever referred in exam** No Yes	313 89	77.86 22.14
**Living situation** Home Hostel	121 281	30.10 69.90
**Food quality satisfaction** No Yes	349 53	86.82 13.18
**Support from Peer** No Yes	91 311	22.63 77.36
**Support from senior** No Yes	109 293	27.11 72.88
**Smoking status** Never Ever	351 51	87.31 12.69
**Nutritional status** Normal Underweight Overweight/obese	234 51 117	58.21 12.69 29.10
**Exercise** Never Rarely Regularly or sometimes	87 174 141	21.64 43.28 37.07
**Sleeping trouble** No Yes	237 165	58.96 41.04
**Tea/coffee consumption per week** 1–3 4–6 7–10 >10	160 68 78 96	39.80 16.92 19.40 23.88
**Fatty food consumption per week** 1–3 4–6 6+	246 96 60	61.19 23.88 14.93
**Family history of IBS** No Yes	320 82	79.60 20.40
**Ever diagnosed with IBS** No Yes	348 54	85.57 15.52

*IBS: Irritable Bowel Syndrome (IBS).

Note: Cells with low frequencies have been merged for further analysis, especially for variables such as age, sex, family status, type of residence, living situation, smoking status, nutritional status, exercise, and fatty food consumption.

### 3.2 Prevalence and subtypes of IBS

The overall prevalence of IBS among the study participants was 22.88 %. Among the participants diagnosed with IBS, 35.87 % were classified as IBS-D (diarrhea-predominant), 26.08 % as IBS-C (constipation-predominant), and 38.04 % as IBS-M (mixed) based on the Rome III criteria (Table 2).

**Table 2:** Prevalence of IBS and distribution of IBS subtypes.

IBS Status	Number of Participants	Prevalence/Percentage
IBS (Overall)	92	22.88 %
**IBS Subtypes**		
IBS-D (Diarrhea-predominant)	33	35.87 %
IBS-C (Constipation-predominant)	24	26.08 %
IBS-M (Mixed)	35	38.04 %

### 3.3 Association of dormitory lifestyle with IBS

The logistic regression analysis revealed a significant association between dormitory lifestyle and the risk of IBS. After adjusting for potential confounders, students residing in hostels were found to have higher odds of IBS compared to those living at home (OR = 2.11, 95 % CI: 1.05–4.25, p < 0.001) (Table 3).

**Table 3:** Correlation of dormitory lifestyle with IBS.

Variables	Unadjusted		Adjusted	
	OR (95 % CI)	p-value	OR (95 % CI)	p-value
Age 17–22 23–28	(Ref.) 1.86 (1.06–3.26)	0.030	(Ref.) 1.39 (0.69–2.82)	0.353
Living situation Home Hostel	(Ref.) 2.43 (1.36–4.10)	0.002	(Ref.) 2.11 (1.45–4.25)	<0.001
Support from senior	(Ref.) 2.39 (1.30–4.39)	0.06	(Ref.) 2.36 (1.05–5.30)	0.05
Support from Peer No Yes	(Ref.) 0.41 (0.23–0.74)	0.062	(Ref.) 0.45 (0.25–1.10)	0.055
Study phase 1st phase 2nd phase 3rd phase 4th phase	(Ref.) 2.64 (1.32–5.27)	0.004	(Ref.) 2.43 (1.59–3.50)	0.002
Exercise Never Rarely Regularly or sometimes	(Ref.) 2.56 (2.31–4.06)	0.004	(Ref.) 2.78 (2.05–4.25)	**0.036**
Fatty food consumption per week 1–3 4–6 6+	(Ref.) 1.03 (0.50–2.10) 2.62 (1.32–5.21)	0.938 0.006	(Ref.) 0.95 (0.39–2.25) 2.98 (1.19–7.42)	0.901 0.03
Academic stress	1.05 (1.02–1.08)	0.001	2.03 (1.99–2.07)	0.002

* Academic stress has been included in the model as a continuous variable.

### 3.4 Establishing temporal precedence

To further support the causal relationship, temporal precedence was established by assessing the duration of dormitory or hostel residence among students with IBS. The results showed that among the students living in dormitories, those who had resided in dormitories for a longer duration had a higher prevalence of IBS than those with a shorter duration of residence (Table 4).

**Table 4:** Prevalence of IBS by duration of dormitory residence.

Duration of Dormitory Residence	Number of Students	Students with IBS	Prevalence of IBS
<1 year	79	16	20.25 %
1–3 years	99	24	24.24 %
>3 years	103	30	29.13 %

### 3.5 Mediating factors for IBS

To further establish the causal mechanism, the study explored potential mediating factors associated with dormitory living that could contribute to the increased risk of IBS. The study identified several other factors related to an increased risk of IBS. Participants aged 23–28 years had higher unadjusted odds of IBS compared to those aged 17–22 years (OR = 1.86, 95 % CI: 1.06–3.26, p = 0.030) (Table 3). Lack of support from seniors (OR = 2.36, 95 % CI: 1.05–5.30, p = 0.05) and being in the second phase of the study (OR = 2.43, 95 % CI: 1.59–3.50, p = 0.002) were also associated with higher odds of IBS after adjustment. Regular exercise appeared to have a protective effect against IBS (OR = 2.11, 95 % CI: 1.05–4.25, p = 0.036), while frequent consumption of fatty foods (6+ times per week) was associated with increased odds of IBS (OR = 2.98, 95 % CI: 1.19–7.42, p = 0.03) (Table 3).

### 3.6 Academic stress and IBS

Academic stress, analyzed as a continuous variable, was a significant predictor of IBS, with higher stress levels associated with increased odds of IBS (OR = 2.03, 95 % CI: 1.99–2.07, p = 0.002) (Table 3). The results showed that students living in dormitories had significantly higher academic stress levels (high stress: 54.44 % vs. 43.78 %, p = 0.035), lower levels of exercise (regularly or sometimes: 48.23 % vs. 51.77 %, p = 0.422), and poorer dietary habits (fatty food consumption 6+ times per week: 53.33 % vs. 46.67 %, p = 0.134) compared to those living outside. The bivariate analysis revealed that academic stress was significantly associated with various factors, including age, type of residence, ever being referred in an exam, lack of support from peers and seniors, smoking status, sleeping trouble, and family history of IBS (Table 5).

**Table 5:** Bivariate analysis for academic stress.

Variables	Academic stress		P value
	Low (%)	High (%)	
**Age** 17–22 23–28	131 (56.22) 77 (45.56)	102 (43.78) 92 (54.44)	**0.035**
**Sex** Female Male	93 (53.76) 115 (50.22)	80 (46.24) 114 (49.78)	0.482
**Family socioeconomic status** Lower or lower-middle Middle Upper or upper-middle	27 (49.09) 122 (51.05) 59 (54.63)	28 (50.91) 117 (48.95) 49 (45.37)	0.755
**Type of residence** Rural or semi-urban Urban	64 (62.75) 144 (48.00)	38 (37.25) 156 (52.00)	**0.010**
**Degree enrolled** BDS MBBS	8 (33.33) 200 (52.91)	16 (66.67) 178 (47.09)	0.063
**Study phase** 1st phase 2nd phase 3rd phase 4th phase	83 (59.29) 38 (52.05) 37 (43.02) 50 (48.54)	57 (40.71) 35 (47.95) 49 (56.98) 52 (51.46)	0.101
**Ever referred in exam** No Yes	171 (54.63) 37 (41.57)	142 (45.37) 52 (58.43)	**0.030**
**Living situation** Home Hostel	55 (45.45) 153 (54.45)	66 (54.55) 128 (45.55)	0.098
**Food quality satisfaction** No Yes	183 (52.44) 29 (54.72)	166 (47.56) 24 (45.28)	0.715
**Support from Peer** No Yes	35 (38.89) 177 (56.91)	55 (61.11) 134 (43.09)	**<0.001**
**Support from senior** No Yes	47 (43.12) 186 (63.48)	62 (56.88) 107 (36.52)	**0.010**
**Smoking status** Never Yes	189 (53.85) 19 (37.25)	162 (46.15) 32 (62.75)	**0.027**
**Nutritional status** Normal Underweight Overweight/obese	131 (55.98) 22 (43.14) 55 (47.01)	103 (44.02) 29 (56.86) 62 (52.99)	0.120
**Exercise** Never Rarely Regularly or sometimes	40 (45.98) 95 (54.60) 73 (51.77)	47 (54.02) 79 (45.40) 68 (48.23)	0.422
**Sleeping trouble** No Yes	138 (58.23) 70 (42.42)	99 (41.77) 95 (57.58)	**0.002**
**Tea/coffee consumption per week** 1–3 4–6 7–10 >10	84 (52.50) 39 (57.35) 38 (48.72) 47 (48.96)	76 (47.50) 29 (42.65) 40 (51.28) 49 (51.04)	0.687
**Fatty food consumption per week** 1–3 4–6 6+	137 (55.69) 43 (44.79) 28 (46.67)	109 (44.31) 53 (55.21) 32 (53.33)	0.134
**Family history of IBS** No Yes	178 (55.63) 30 (36.59)	142 (44.38) 52 (63.41)	**0.002**
**Ever diagnosed with IBS** No Yes	181 (52.01) 27 (50.00)	167 (47.99) 27 (50.00)	0.783

## 4 Discussion

This study examined the frequency of irritable bowel syndrome (IBS) and its correlation with living in dormitories among medical students in Bangladesh. The prevalence of Irritable Bowel Syndrome (IBS) was determined to be 22.88 %, aligning with earlier findings indicating that IBS impacts around 10–25 % of the general population [26,27]. The subjects diagnosed with IBS were distributed as follows: 35.87 % had diarrhea-predominant IBS (IBS-D), 26.08 % had constipation-predominant IBS (IBS–C), and 38.04 % had mixed IBS (IBS-M), according to the Rome III criteria.

An essential discovery of this study was the robust correlation between living in a dormitory and a heightened susceptibility to IBS. After controlling for potential variables that could affect the results, it was discovered that students residing in hostels had 2.11 times greater chances of having IBS compared to those who lived at home. This result was statistically significant, with a 95 % confidence interval of 1.05–4.25 and a p-value of less than 0.001. This finding aligns with other research that has established a connection between living in dormitories and various health problems, such as gastrointestinal diseases [20,21]. Significantly, the occurrence of IBS rose in correlation with the length of time spent living in a dormitory, supporting the temporal precedent concept. Students who lived in dorms for over three years had a significantly greater occurrence of Irritable Bowel Syndrome (IBS) at a rate of 29.13 %, in contrast to those who lived in dormitories for less than one year, with a prevalence of 20.25 %. The temporal link between dormitory living and the development of IBS provides additional evidence supporting a causal association. The study investigated potential factors that could mediate the higher risk of Irritable Bowel Syndrome (IBS) among individuals living in dormitories. Students residing in hostels exhibited a notably higher prevalence of academic stress (54.44 % vs. 43.78 %, p = 0.035), lower levels of physical activity (48.23 % vs. 51.77 %, p = 0.422), and inferior dietary habits, including frequent consumption of fatty foods (53.33 % vs. 46.67 %, p = 0.134), in comparison to their peers residing elsewhere [[28], [29], [30], [31]].

Mediation analysis demonstrated that the impact of living in a dormitory on IBS was partly mediated by academic stress, levels of physical activity, and dietary choices. This discovery implies that living in a dormitory may heighten the likelihood of developing Irritable Bowel Syndrome (IBS) due to its influence on these parameters, offering proof for a credible cause-and-effect mechanism. In addition, the study discovered many additional risk variables that are linked to a higher likelihood of developing IBS. Individuals between the ages of 23 and 28 had a greater likelihood of having IBS compared to those between the ages of 17 and 22 (OR = 1.86, p = 0.030). This could be due to the combined impact of stress and lifestyle factors that accumulate over time. The study found that a lack of support from seniors (OR = 2.36, p = 0.05) and being in the second phase of the study (OR = 2.43, p = 0.002) were both related to higher chances of experiencing Irritable Bowel Syndrome (IBS). This could be attributed to the heightened academic pressures and stress typically occurring during these periods [29]. Engaging in regular exercise was found to have a protective effect against IBS (OR = 2.11, p = 0.036), which aligns with earlier studies indicating that physical activity might reduce symptoms of IBS and enhance overall gut health [32]. In contrast, regularly eating fatty foods more than six times per week was linked to a higher likelihood of developing IBS (odds ratio = 2.98, p = 0.03). This emphasizes the significance of maintaining a well-balanced diet for managing IBS.

The study found that academic stress was a strong indicator of irritable bowel syndrome (IBS), with higher levels of stress being linked to a greater likelihood of developing IBS (odds ratio = 2.03, p = 0.002). This discovery is consistent with the widely recognized connection between stress and the formation of functional gastrointestinal diseases, such as IBS [33,34]. The bivariate analysis demonstrated a significant correlation between academic stress and several factors, including age, type of residence, lack of support from peers and seniors, smoking status, sleeping trouble, and family history of IBS. It highlights the multifactorial nature of IBS and the intricate interaction between lifestyle, psychological, and environmental factors.

In conclusion, this study provides compelling evidence establishing a causal link between residing in dormitories and an increased susceptibility to Irritable Bowel Syndrome (IBS) among university students. The findings underscore the need to promote healthy lifestyles, including the adoption of stress management strategies, frequent participation in physical exercise, and adherence to a well-balanced diet in the context of university hostels. Implementing interventions that specifically address these modifiable risk factors can mitigate the effects of IBS and improve the overall well-being of students residing in dormitories. Further longitudinal research is required in the future to have a more comprehensive understanding of the causal mechanisms underlying the association between living in dorms and irritable bowel syndrome (IBS). These trials should also evaluate the efficacy of targeted medicines in mitigating the risk and managing the symptoms of IBS in this group.

## 5 Strengths and limitations

To the best of our knowledge, this is the first study in Bangladesh to assess the prevalence and associated factors of IBS in medical students, adding to the existing literature gap. Moreover, data collection involved a questionnaire covering various variables, including socio-demographics, lifestyle, family history, academic stress, and diagnostic criteria for IBS. This comprehensive approach allowed for a more in-depth analysis.

On the other hand, the study's cross-sectional design allows for the identification of associations but not causation. Longitudinal studies would be needed to establish causal relationships. The study relied on self-reported data, subject to recall and social desirability biases. Participants may underreport sensitive information. While the study examined several factors associated with IBS, there may be unmeasured confounding variables that should have been accounted for in the analysis. One limitation is using the Rome III criteria, while Rome IV criteria are more accurate at diagnosing IBS. However, Rome III criteria have been widely used in large-scale epidemiological studies, making it a well-validated and widely accepted standard for IBS prevalence. Our results can be compared to global and South Asian IBS prevalence research using the Rome III criteria.

Despite these limitations, this study provides valuable insights into the prevalence and associated factors of IBS among medical students in Bangladesh, highlighting the need for further research and targeted interventions to support their well-being.

## 6 Conclusion

This study shows that Bangladeshi medical students had a high rate of IBS. It emphasizes the impact of dorm life, academic stress, and poor diet. Global estimates put the prevalence of irritable bowel syndrome (IBS) at 22.88 %, demonstrating its considerable impact on this group. The study's novel finding is that dormitories increase IBS risk, even after controlling for potential confounders. Hostel students had 2.11 times the risk of IBS compared to those at home. There was also a direct association between dormitory time and IBS risk, showing a cause-and-effect relationship. The study discovered intervening characteristics that could elucidate the dormitory-IBS causal link. The hostel residents had high academic stress, low physical activity, and unhealthy diets, including eating fatty foods. Mediation analysis showed that these traits partially mediated the effect of dormitory living on IBS risk, revealing the underlying mechanisms.

The findings highlight the need to address changing risk factors to improve medical students' lives, particularly those in dorms. Stress management, exercise, and proper eating can lessen IBS in this group. Improved dormitory accommodations and support structures can reduce risk. This study provides important insights, but further longitudinal research is needed to establish stronger causal links and evaluate targeted therapy for IBS in Bangladeshi medical students. The extensive prevalence of IBS and its accompanying disorders must be managed to protect these prospective healthcare practitioners' health and education.

## Funding

N/A.

## Data availability statement

Available upon request.

## CRediT authorship contribution statement

**Tirthom Das:** Writing – review & editing, Writing – original draft, Supervision, Project administration, Data curation, Conceptualization. **Fahmida Hoque Rimti:** Writing – review & editing, Writing – original draft, Methodology. **Hossain Ahmed Fahid:** Writing – original draft, Resources, Investigation. **Nabil Ahmed Uthso:** Visualization, Software, Formal analysis. **Mohammad Delwer Hossain Hawlader:** Writing – review & editing, Supervision, Resources.

## Declaration of competing interest

The authors declare that they have no known competing financial interests or personal relationships that could have appeared to influence the work reported in this paper.
