# Supplementary material for: Prevalence of IBS and its association with academic stress and dormitory lifestyle among medical students of Bangladesh: A cross-sectional study
Source: Heliyon. 2024 Aug 13;10(16):e36259. doi: 10.1016/j.heliyon.2024.e36259 (PMC11367496; doi:10.1016/j.heliyon.2024.e36259)
Supplement: Multimedia component 2 [file mmc2.docx]

**APPENDIX-1**

| **QUESTIONS** |
| --- |
| Name |
| Age.   1. 17-19 2. 20-22 3. 23-25 4. 26-28 |
| Sex   1. Female 2. Male 3. Prefer not to say |
| 1. Family status. Lower socioeconomic class. 2. Lower-Middle socioeconomic class. 3. Middle socioeconomic class. 4. Upper-Middle Socioeconomic class. 5. Upper socioeconomic class. |
| Habitat   1. Urban 2. Semi-urban 3. Rural |

**APPENDIX-2**

| **QUESTIONS** |
| --- |
| Which course are you enrolled in?   1. MBBS 2. BDS |
| Which phase are you currently studying in?   1. 1st phase(1st-2nd year) 2. 2nd phase(3rd year) 3. 3rd phase(4th year) 4. 4th phase(5th year) |
| Have you ever been referred in a professional examination?   1. Yes 2. No |
| What is your living situation?   1. At home, 2. At Hostel 3. At relative’s home |
| If you answered 2 to the previous question, Are you satisfied with the food quality provided in the hostel?   1. Yes 2. No |
| If you answered 2 to the previous question, do you think you are supported by your peers(friends/batchmate) in the hostel?   1. Yes 2. No |
| If you answered 2 to the previous question, do you think you are supported by your seniors in the hostel?   1. Yes 2. No |

**APPENDIX-3**

| **QUESTIONS** |
| --- |
| Do you smoke?   1. Regularly 2. Sometimes 3. Rarely 4. Never |
| What is your nutritional status?   1. Underweight 2. Normal 3. Overweight 4. Obese |
| How often do you excercise?   1. Regularly 2. Sometimes 3. Rarely 4. Never |
| Do you have trouble sleeping?   1. Yes 2. No |
| How often do you consume tea or coffee per week?   1. 1-3 2. 4-6 3. 7-10 4. >10 |
| How often do you consume fatty foods per week?   1. 1-3 2. 4-6 3. 7-10 4. >10 |

**APPENDIX-4**

| Do you have a family history of IBS?  1)Yes  2)No |
| --- |
| Have you been diagnosed with IBS in the past?  1)Yes  2)No |
| At least 12 weeks or more, which need not be consecutive, in the preceding 12 months, present abdominal discomfort or pain that has two out of the following three features:  · Relieved by defaecation  · Onset associated with a change in stool frequency  · Onset associated with a change in the form of stool |
| Symptoms that cumulatively support the diagnosis of IBS:  · Abnormal stool frequency (may be defined as greater than 3 bowel movements per day and less than 3 bowel movements per week)  · Abnormal stool form (lumpy/hard or loose/watery stool)  · Abnormal stool passage (straining, urgency, or feeling of incomplete evacuation)  · Passage of mucus  · Bloating or feeling of abdominal distension. |

**APPENDIX- 5**

| **QUESTIONS** | Strongly agree | Agree | Neutral | Disagree | Strongly Disagree |
| --- | --- | --- | --- | --- | --- |
| I’m confident that I will be a successful student | 5 | 4 | 3 | 2 | 1 |
| I Am confident that I will be a successful in my future career | 5 | 4 | 3 | 2 | 1 |
| I can make academic decisions easily | 5 | 4 | 3 | 2 | 1 |
| The time allocated to my academic studies is enough | 5 | 4 | 3 | 2 | 1 |
| I have enough time to relax after work | 5 | 4 | 3 | 2 | 1 |
| My teachers are critical of my academic performance | 5 | 4 | 3 | 2 | 1 |
| I fear failing courses this year | 5 | 4 | 3 | 2 | 1 |
| I think that my worry about examinations is weakness of character | 5 | 4 | 3 | 2 | 1 |
| Teachers have unrealistic expectations of me | 5 | 4 | 3 | 2 | 1 |
| The size of the curriculum (workload) is excessive | 5 | 4 | 3 | 2 | 1 |
| I believe that the amount of work assignment is too much | 5 | 4 | 3 | 2 | 1 |
| I Am unable to catch up if getting behind the work | 5 | 4 | 3 | 2 | 1 |
| The unrealistic expectations of my parents stresses me out | 5 | 4 | 3 | 2 | 1 |
| competition with my peers for grades is quite intense | 5 | 4 | 3 | 2 | 1 |
| The examination questions are usually difficult | 5 | 4 | 3 | 2 | 1 |
| Examination time is short to complete the answers | 5 | 4 | 3 | 2 | 1 |
| Examination times are very stressful to me out | 5 | 4 | 3 | 2 | 1 |
| Even if I pass my exams, am worried about getting a job | 5 | 4 | 3 | 2 | 1 |
