# Supplementary figures and images for: Prevalence of IBS and its association with academic stress and dormitory lifestyle among medical students of Bangladesh: A cross-sectional study
Source: Heliyon. 2024 Aug 13;10(16):e36259. doi: 10.1016/j.heliyon.2024.e36259 (PMC11367496; doi:10.1016/j.heliyon.2024.e36259)

Strobe Flow Diagram


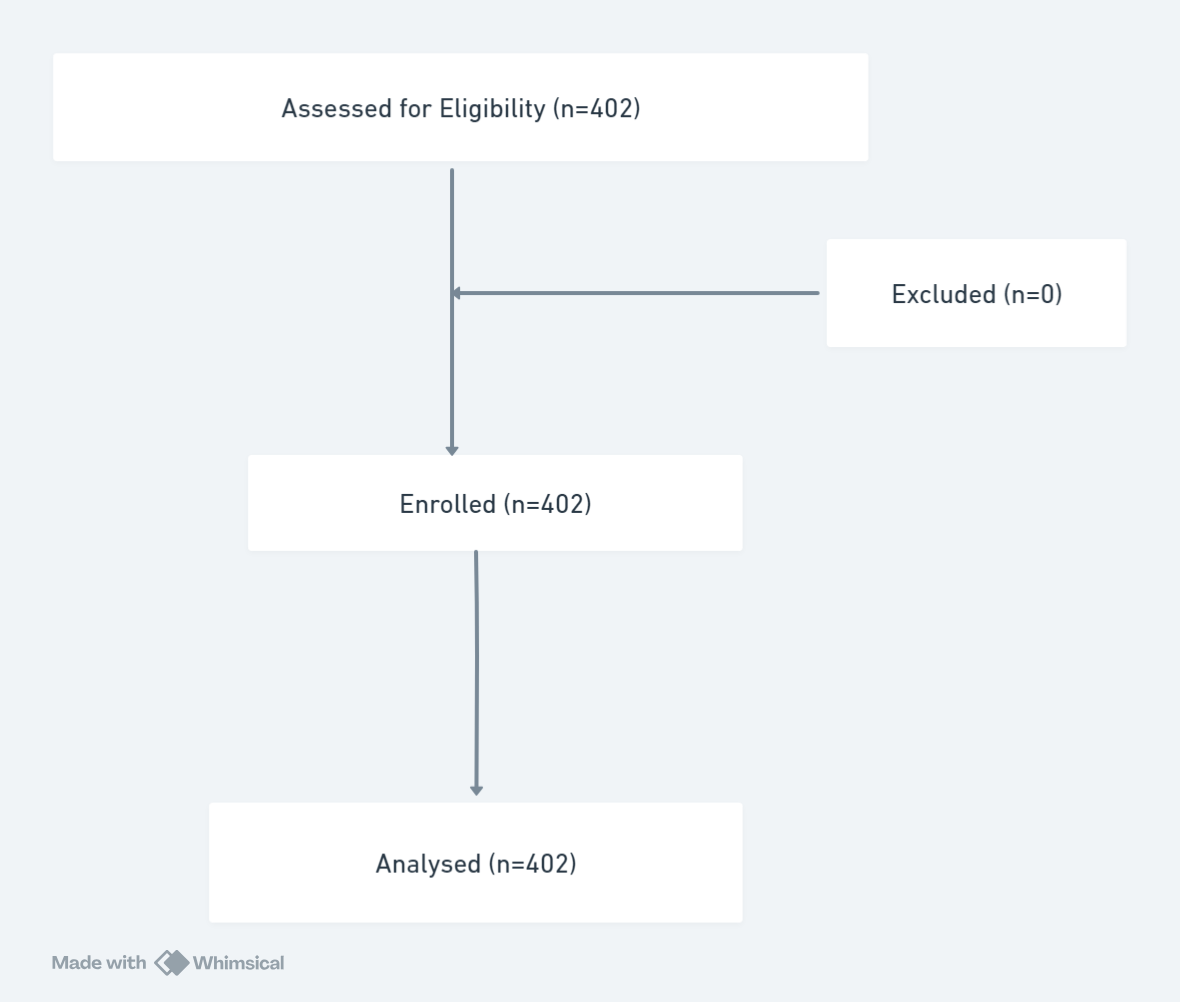

Supplement: Multimedia component 3 [file mmc3.docx]
